# Synergistic effects of collagen membrane and mineral trioxide aggregate on odontogenic differentiation and mineralization of human dental pulp stem cells: an in vitro study

**DOI:** 10.1186/s12903-026-07960-0

**Published:** 2026-03-20

**Authors:** Nermine Hassan, Nashwa El-Khazragy, Amir Hafez Ibrahim

**Affiliations:** 1 Department of Endodontics, Faculty of Dentistry, Cairo University, Cairo, 11553 Egypt; 2 Department of Clinical Pathology-Hematology, Ain Shams Medical Research Institute (MASRI), Faculty of Medicine, Ain Shams University, Cairo, 11566 Egypt; 3 Department of Conservative Dentistry, Faculty of Dentistry, Cairo University, Cairo, 11553 Egypt

**Keywords:** Human dental pulp stem cells, Collagen, Mineral Trioxide Aggregate, Odontogenic differentiation, Gene expression, Mineralization

## Abstract

**Background:**

Human dental pulp stem cells (hDPSCs) are oral-derived mesenchymal stem cells with high proliferative capacity and odontogenic differentiation potential, making them relevant for dental pulp and dentin–pulp complex regeneration. This in vitro study evaluated the effects of collagen membrane and mineral trioxide aggregate (MTA) conditioned media, individually and in combination, on hDPSC viability, odontogenic differentiation, and mineralization.

**Methods:**

hDPSCs were isolated, expanded, and characterized by flow cytometry, then cultured in osteogenic differentiation medium and treated with conditioned media derived from collagen membrane, MTA, or their combination. Cell viability was assessed after 48 h using the MTT assay. Odontogenic differentiation was evaluated after 7 days by alkaline phosphatase activity and Alizarin Red S staining, while late-stage differentiation was analyzed after 21 days by quantitative real-time PCR of odontogenic-associated genes (*RUNX2*, *DMP1*, and *BMP2*).

**Results:**

Collagen membrane–conditioned medium moderately increased cell viability but showed limited effects on odontogenic differentiation. In contrast, MTA-conditioned medium significantly enhanced differentiation and mineral deposition. The combined collagen membrane + MTA–conditioned medium produced the most pronounced effects, significantly increasing alkaline phosphatase activity, odontogenic gene expression, and mineralized matrix formation.

**Conclusion:**

These findings suggest that soluble factors released from collagen membrane and MTA act synergistically to promote odontogenic differentiation of hDPSCs in vitro, with potential relevance to dental pulp regenerative strategies.

**Graphical Abstract:**

**Figure:**
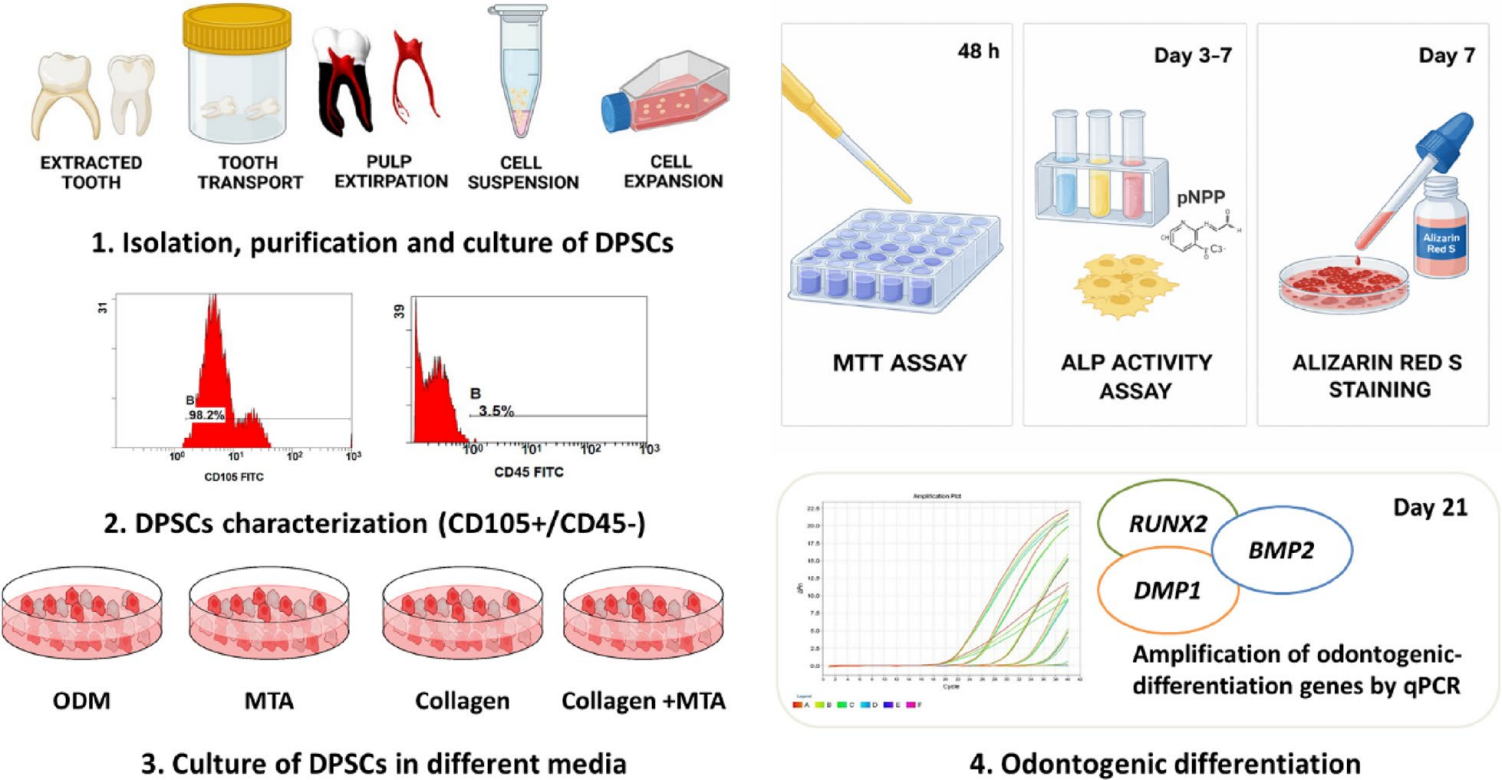
Experimental workflow: Human dental pulp stem cells (hDPSCs) were isolated, characterized, and cultured in osteogenic differentiation medium (ODM) alone or supplemented with collagen, MTA, or their combination. Cell viability was assessed at 48 h, odontogenic differentiation at 7 days (ALP activity and Alizarin Red S staining), and gene expression (RUNX2, DMP1, BMP2) at 21 days by qPCR.

## Introduction

The preservation of dental pulp is crucial for maintaining tooth vitality, especially when exposed due to trauma or decay [1]. The dental pulp, often referred to as the “heart” of the tooth, contains nerves and blood vessels necessary for tooth survival [2]. When the pulp is exposed, it is susceptible to infection and necrosis, which can ultimately lead to tooth loss if not properly managed [3]. Vital pulp therapy is a key treatment strategy to preserve pulp health by protecting and regenerating pulp tissue [4]. This therapy often involves the use of pulp capping agents, which are materials designed to protect the pulp, promote healing, and facilitate dentin bridge formation over exposed pulp tissue [5].

Several pulp capping agents have been developed, with their effectiveness evaluated based on their ability to induce odontogenic differentiation and protect the pulp from infection [6]. These agents are typically divided into two categories: conventional materials like calcium hydroxide and biomimetic materials such as mineral trioxide aggregate (MTA) [7, 8], Biodentine [9], and bioceramic pastes [10, 11]. MTA, a calcium silicate-based material, has gained popularity due to its ability to stimulate odontogenic differentiation in human dental pulp stem cells (hDPSCs) and promote the formation of dentin bridges [3]. It is known for its bioactivity, sealing properties, and biocompatibility. Unlike calcium hydroxide, which can induce a significant inflammatory response, MTA causes only mild, transient inflammation and minimal pulp necrosis, making it a preferred material for many clinicians [12].

Oral-derived mesenchymal stem cells (MSCs), including dental pulp stem cells and other craniofacial MSC populations, exhibit high proliferative capacity, multilineage differentiation potential, and strong odontogenic commitment, making them particularly suitable for dental regenerative applications. Moreover, these cells possess immunomodulatory and tissue-regenerative properties that support their growing relevance in dental and craniofacial tissue engineering strategies [13, 14].

Collagen membranes, which are biodegradable and exhibit low immunogenicity, have recently emerged as a promising material in pulp capping. Collagen’s natural biocompatibility and ability to support tissue repair processes, Collagen-based materials have been widely investigated for pulp-related therapies [11] Previous studies have reported that collagen membranes can enhance dentin bridge formation and maintain pulp vitality, offering several advantages over conventional materials like calcium hydroxide [15]. In addition, collagen-derived components have been shown to support cell proliferation and odontogenic differentiation, as well as promote mineral deposition, by providing a microenvironment that resembles aspects of the native extracellular matrix (ECM) of dental pulp tissue [16]. Additionally, collagen membranes can enhance mineralization and direct the deposition of hydroxyapatite crystals, mimicking the natural extracellular matrix (ECM) of dental pulp [17].

Despite these biological advantages, collagen membranes exhibit limitations related to mechanical strength and long-term stability, which may restrict their performance as standalone scaffolds in pulp regeneration [18]. To address these limitations, strategies such as material cross-linking or combination with bioactive materials have been explored [19]. Mineral trioxide aggregate (MTA), a calcium silicate–based material with well-established biocompatibility and sealing ability, has been widely used in pulp capping procedures. Combining collagen-based materials with MTA has been proposed as a means to enhance biological performance by integrating the favorable biological properties of collagen with the odontogenic inductive potential of MTA [20]. Experimental evidence suggests that MTA-derived bioactive factors promote odontogenic differentiation and mineralized tissue formation, while collagen-based materials may modulate the cellular microenvironment and influence the biological activity of MTA [19]. However, the mechanisms underlying the potential synergistic effects of collagen and MTA [21]. However, the mechanisms underlying the potential synergistic effects of collagen and MTA, particularly through soluble factors rather than direct scaffold–cell interactions, remain incompletely understood and warrant further investigation [20, 21].

Despite the promising results, the synergistic effects of collagen and MTA in pulp capping therapy are not fully understood, and further investigation is needed. Research into the molecular mechanisms behind the interaction of these materials will be crucial in optimizing their use in clinical practice. Understanding how collagen and MTA work together to enhance pulp healing and regeneration could lead to better pulp capping therapies and more effective treatment options for patients with exposed dental pulp.

The primary objectives of this study were to evaluate the synergistic effects of MTA and collagen, both individually and in combination, on the proliferative potential of hDPSCs. Additionally, the study aimed to assess the ability of these materials to promote odontogenic differentiation at both cellular and genetic levels.

## Materials & methods

### Materials

All cell culture reagents were purchased from Gibco, Thermo Fisher Scientific. The osteogenic differentiation medium (ODM) was prepared using α-MEM (Gibco, Thermo Fisher Scientific, Cat. No. 32561029), supplemented with 100 nM dexamethasone (Sigma-Aldrich, Cat. No. D4902), 200 µM ascorbic acid (Sigma-Aldrich, Cat. No. A8960), and 10 mM β-glycerophosphate (Sigma-Aldrich, Cat. No. G9891), along with 1% penicillin-streptomycin-amphotericin B (Gibco, Thermo Fisher Scientific, Cat. No. 15140122). The Alkaline Phosphatase (ALP) Assay Kit (Sigma-Aldrich, Cat. No. MAK447) and Alizarin Red S (ARS) Kit (Sigma-Aldrich, Cat. No. 0016553000) were used to assess odontogenic differentiation and mineralization, respectively. For PCR analysis, the QuantiTect Reverse Transcription Kit (Qiagen, Cat. No. 205310) and the QuantiTect SYBR Green PCR Kit (Qiagen, Cat. No. 204141) were utilized. The used primers are: oligo-specific primer sequences [Hs_RUNX2_1_SG, assay ID: QT00020517, Hs_DMP1_1_SG QuantiTect Primer Assay, assay ID: QT00022078, and Hs_BMP_vc.2_SG, assay ID: QT00024535 QuantiTect Primer Assay, cat no: 249900 and the β-actin (Hs_ACTB), ID: QT000954231 primer assay “as housekeeper gene”. The tested materials used in the study were MTA Angelus White, a bioceramic reparative cement composed of tricalcium silicate, dicalcium silicate, tricalcium aluminate, calcium oxide, and calcium tungstate (Angelus, Londrina, PR, Brazil), and Colla, a resorbable collagen membrane derived from bovine collagen (Medpark, Co., Ltd., Republic of Korea).

### Isolation, purification, and culture of human dental pulp stem cells

**Fig. 1:**
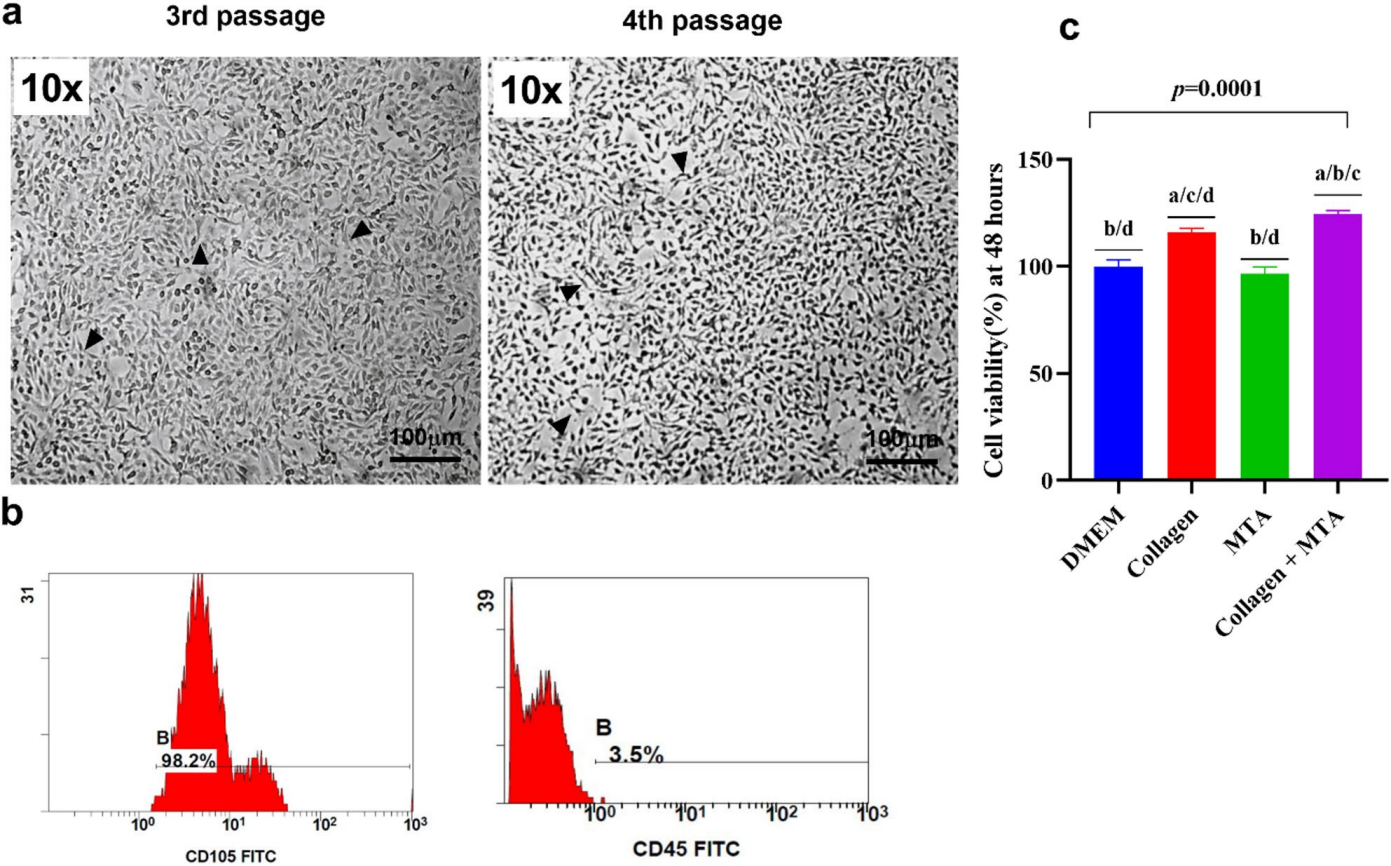
Characterization and Effects of Biomaterials on DPSCs: (**a**) hDPSCs at third and fourth passage showing typical well-differentiated morphology with flattened spindle-shaped cells and hyperchromatic nuclei (20× magnification, scale bar: 20 µm). (**b**) Flow cytometry dot plots for DPSCs stained with CD105-FITC and CD45, showing high CD105 expression (98.2%) and low CD45 expression (3.5%), confirming the purity of DPSCs. (**c**) Bar graph comparing DPSC viability after 48 hours of treatment with Collagen, MTA, and Collagen + MTA, showing significantly higher viability in the Collagen + MTA group. Data are presented as mean ± SD, with statistical significance determined by ANOVA and Tukey's post-hoc test (*p* < 0.05). a: statistical significance compared to cells cultured in DMEM (*p*>0.05), b: statistical significance compared to cells cultured in media supplemented with collagen (*p*>0.05), c: statistical significance compared to cells cultured media supplemented with MTA (*p*>0.05), d: statistical significance compared to cells cultured media supplemented with Collagen + MTA (*p*>0.05)

Human dental pulp stem cells (hDPSCs) were isolated from freshly extracted human premolars obtained from healthy donors (*n* = 3), with prior informed consent and ethical approval. The study was approved by The Ethics Committee of Cairo University’s Faculty of Dentistry (approval no: 19/10/22) and conducted in accordance with the Declaration of Helsinki and its most recent modification, in accordance with applicable science and human subjects’ regulations. The teeth were immediately placed in sterile phosphate-buffered saline (PBS) with antibiotics, antimycotics, and 1% DMSO as a temporary preservation medium. After cleaning the teeth in PBS to remove surface impurities, the pulp chamber was exposed, and the dental pulp tissue was carefully extracted. The tissue was minced and enzymatically digested with collagenase type I and dispase to generate a single-cell suspension, followed by centrifugation. The resulting cell pellet was resuspended in *Dulbecco’s Modified Eagle’s Medium/Nutrient Mixture* F-12 DMEM/F12 medium supplemented with fetal bovine serum (FBS) and antibiotics. The cell suspension was filtered through a 70-µm strainer, seeded into T25 culture flasks, and incubated in a humidified environment at 37 °C with 5% CO2. Adherent fibroblast-like cells characteristic of hDPSCs were observed within 5–7 days. Non-adherent cells were removed by changing the culture medium every two days. The cells were then passaged using trypsin-EDTA and cultured until reaching 80% confluence for downstream analysis (Fig. 1a). Cells from passage 4 were used for experiments, as they exhibit greater stability than earlier passages [22, 23].

### Characterization of hDPSCs

The phenotypic characterization of isolated hDPSCs was performed using multiparametric flow cytometry. The cells were stained with CD45-FITC and CD105-FITC antibodies. The cell suspension, adjusted to a concentration of 10⁶ cells/mL, was centrifuged, washed with PBS, and incubated with the antibodies for 45 min at 4 °C. After incubation, the cells were washed and resuspended in binding buffer. Flow cytometric analysis was conducted using a *NAVIOS EX 10-color flow cytometer (Beckman Coulter)*, and data were analyzed with *Navios software*. The staining was performed with CD105 (MEM-226) FITC and CD45 (30-F11) FITC monoclonal antibodies (Fig. 1b) from Thermo Fisher Scientific [24].

### Preparation of material-conditioned media

Material-conditioned media were prepared to evaluate the biological effects of soluble factors released from collagen membrane and mineral trioxide aggregate (MTA), without direct material–cell contact.

For MTA-conditioned medium, MTA powder was prepared according to the manufacturer’s instructions and placed into sterile silicone molds (5 mm diameter × 2 mm thickness). The material was allowed to set for 24 h at 37 °C and 100% humidity under sterile conditions. After setting, the MTA discs were sterilized by ultraviolet (UV) irradiation for 30 min on each side.

Collagen membrane was supplied in sheet form and was not mixed with unset MTA. Instead, collagen membranes were aseptically cut into standardized pieces of comparable surface area, sterilized by UV irradiation, and processed separately to generate collagen membrane–conditioned medium.

For conditioned medium preparation, individual MTA discs, collagen membrane pieces, or a combination of both were immersed in 15 mL of DMEM culture medium in sterile Falcon tubes and incubated for 24 h at 37 °C to allow the release of soluble bioactive components. Following incubation, the suspensions were centrifuged at 4000 × g for 15 min to remove particulate matter. The resulting supernatants were collected, filtered if necessary, aliquoted into sterile tubes, and stored at − 20 °C until use in subsequent cell culture experiments [25].

### Assessment of cell proliferation and viability of DPSCs cultured with different dental materials

Dental pulp stem cells (DPSCs) were cultured in a 96-well plate at a density of 1 × 10^4 cells per well and incubated for 24 h at 37 °C in a humidified atmosphere containing 5% CO₂ to allow cell attachment. Cells were maintained in DMEM supplemented with 10% fetal bovine serum (FBS) and 1% penicillin-streptomycin-amphotericin B (PSA). After the initial incubation period, the culture medium was replaced with DMEM supplemented with conditioned media derived from collagen membrane, or mineral trioxide aggregate (MTA), or a combination of collagen membrane and MTA. Cells cultured in DMEM alone served as the control group. Conditioned media were prepared as described in "Preparation of material-conditioned media" section and filtered prior to use to remove particulate material. Cell viability and proliferation were assessed after 48 h of treatment using the Vybrant ^®^ MTT Cell Proliferation Assay Kit (Thermo Fisher Scientific), according to the manufacturer’s instructions. Briefly, MTT reagent was added to each well and incubated for 4 h at 37 °C, allowing the formation of formazan crystals. The supernatant was then removed, and the crystals were solubilized. Absorbance was measured at 570 nm using a microplate spectrophotometer, and cell viability was expressed as a percentage relative to the control group. Cell viability and proliferation were assessed after 48 h of treatment using the Vybrant^®^ MTT Cell Proliferation Assay Kit (Thermo Fisher Scientific), according to the manufacturer’s instructions. Briefly, MTT reagent was added to each well and incubated for 4 h at 37 °C, allowing the formation of formazan crystals. The supernatant was then removed, and the crystals were solubilized. Absorbance was measured at 570 nm using a microplate spectrophotometer, and cell viability was expressed as a percentage relative to the control group [26].

### Odontogenic differentiation and mineralization of h DPSCs

#### ALP activity assay and alizarin red s mineralization assays

Human dental pulp stem cells (hDPSCs) were seeded in 6-well culture plate, at a density of 1 × 10³ cells per well and cultured in osteogenic differentiation medium (ODM) consisting of α-MEM supplemented with dexamethasone, ascorbic acid, and β-glycerophosphate. After cell attachment, cultures were treated with conditioned media derived from collagen membrane, mineral trioxide aggregate (MTA), or a combination of collagen membrane and MTA. Cells cultured in ODM alone served as the control group.

Early odontogenic differentiation was evaluated by measuring alkaline phosphatase (ALP) activity after 7 days of treatment using an ALP assay kit (Sigma-Aldrich, USA), according to the manufacturer’s instructions. Briefly, cells were incubated with p-nitrophenyl phosphate substrate, and absorbance was measured at 405 nm using a microplate reader.

To evaluate mineralization [27], Alizarin Red S staining was performed on days 7 and 14 to detect calcium deposition in the extracellular matrix. The dye was then dissolved, and photometric quantification of the calcium-bound stain was performed [28]. Both assays followed the manufacturer’s instructions, and all tests were carried out in triplicate to ensure reproducibility.

#### Assessment of odontogenic differentiation in DPSCs using PCR

For gene expression analysis, hDPSCs were harvested after 21 days of treatment under the same conditioned media and control conditions described above. Total RNA was isolated from harvested DPSCs using the RNeasy^®^ Mini Kit (Qiagen, Germany). The concentration and purity of the cDNA were measured using a Qubit fluorometer (Thermo Fisher, USA) to ensure accurate quantification for subsequent PCR analysis.

For reverse transcription, the cDNA synthesis was performed using the QuantiTect Reverse Transcription Kit (Qiagen, Germany). Gene expression analysis focused on Odontogenic differentiation markers, including *RUNX2*, *DMP1*, and *BMP2*, using the QuantiTect Syber Green PCR Kit (Qiagen, Germany) and specific primers. β-actin served as the housekeeping gene, and gene expression levels were normalized using the 2∆∆Ct method. The analysis was conducted using a 5-plex Rotor-Gene PCR Analyzer [29].

### Statistical methods

All statistical analyses were performed using GraphPad Prism version 9 (GraphPad Software, San Diego, CA, USA). Data were first assessed for normality and homogeneity of variance before conducting any analysis. A one-way analysis of variance (ANOVA) was used to identify overall differences among multiple groups, followed by Tukey’s post-hoc test for pairwise comparisons. Data are presented as mean ± standard deviation, with a p-value < 0.05 considered statistically significant. Graphs were generated using Prism’s built-in visualization tools to ensure consistent formatting and accurate representation of the data.

## Results

### Phenotypic characterization of DPSCs

The DPSCs at (P3–P4) demonstrated typical spindle-shaped morphology and a mesenchymal phenotype with 98.2% CD105 positivity and only 3.5% CD45 expression, fulfilling the minimal characterization criteria recommended by the International Society for Cellular Therapy(ISCT) (Fig. 1a and b).

### Comparative proliferative effects of collagen and MTA on DPSCs

The results showed a clear hierarchy in the proliferative potential of DPSCs based on the material used. Untreated cells in DMEM control exhibited near-baseline viability, while MTA alone resulted in a slightly lower mean viability of 96.6%, indicating no significant enhancement but also no impairment of cell survival. Collagen, on the other hand, significantly increased viability to 116%, demonstrating its pro-growth effect. The combination of Collagen + MTA showed the highest viability at 124%, suggesting a synergistic effect that enhances the bioactivity of MTA and creates a more favorable environment for DPSC proliferation. ANOVA test confirmed significant overall group differences (*p* = 0.0001), with post-hoc Tukey comparisons revealing that Collagen + MTA significantly outperformed all other groups. Notably, the combination’s effect was substantially greater than that of MTA alone, which showed no significant difference from the DMEM control. Results are presented in Table 1; Fig. 1c.

**Table 1:** Descriptive statistics of cell viability percentage in the studied groups after 48 h

Group	mean ± SD	Range	*p*-value
DMEM	100 ± 3.10	97.1–103	
Collagen	116 ± 1.9	114–118	0.0003 ^a/c/d^
MTA	96.6 ± 3.05^b/d^	94.6–100	0.4156 ^b/d^
Collagen + MTA	124 ± 1.70^a/b/c^	123–126	0.0001 ^a/b/c^

*F*: statistical value of ANOVAtest, *ANOVA* Analysis of variances test, *DPSCs* Dental pulp- derived mesenchymal stem cells, *DMEM* Dulbecco’s Modified Eagle Medium (DMEM), *MTA* Mineral Trioxide Aggregate

a: statistical significance compared to the DPSCs “untreated cells”* (p *< 0.05), b: statistical significance compared to the Collagen group* (p < *0.05), c: statistical significance compared to the MTA group* (p <* 0.05), d: statistical significance compared to the Collagen + MTA group* (p <* 0.05*) *

### Odontogenic differentiation of DPSCs induced by collagen, MTA, and their combination (Table 2; Fig. 2)

**Fig. 2:**
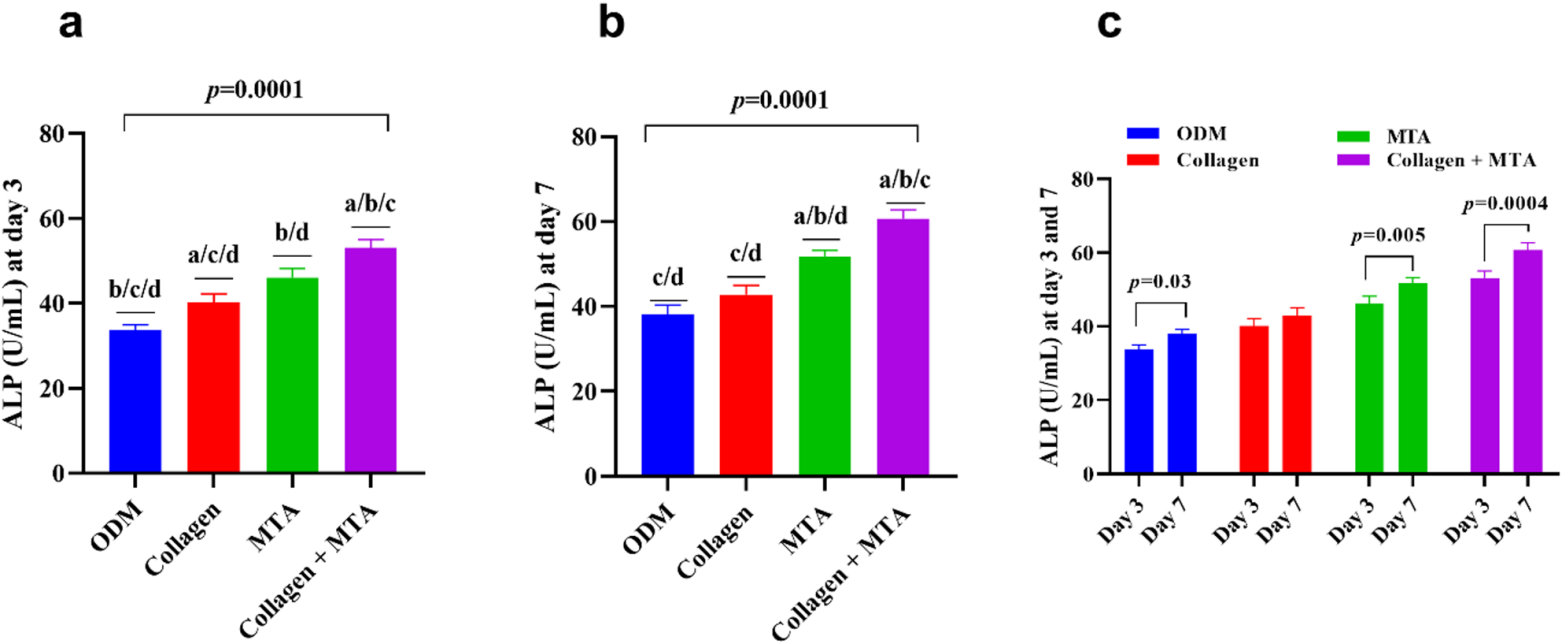
Osteogenic differentiation of DPSCs cultured with ODM, Collagen, MTA, and Collagen + MTA for 3 and 7 days. Bar graphs (**a**, **b**) display ALP activity (U/mL) after 3 days and 7 days of exposure, showing progressively increasing osteogenic responses from Collagen to MTA to Collagen + MTA. Panel (**c**) presents the correlation between ALP activity at day 3 and day 7 for each treatment condition. Error bars represent standard deviations. Statistical significance was assessed using one-way ANOVA followed by Tukey’s post-hoc test. ^a^: statistical significance compared to cells cultured in DMEM (*p* > 0.05), ^b^: statistical significance compared to cells cultured in media supplemented with collagen (*p* > 0.05), ^c^: statistical significance compared to cells cultured media supplemented with MTA (*p* > 0.05), ^d^: statistical significance compared to cells cultured media supplemented with Collagen + MTA (*p* > 0.05). Abbreviations: DPSCs: Dental pulp-derived mesenchymal stem cells, ODM: Osteogenic differentiation medium, MTA: Mineral Trioxide Aggregate, ALP: Alkaline phosphatase.

The odontogenic differentiation of DPSCs showed a clear, progressive enhancement depending on the biomaterial used, with a consistent hierarchy observed at both early and late time points. Collagen alone moderately increased ALP activity compared to the baseline osteogenic medium, while MTA induced a stronger stimulatory effect due to its osteoinductive properties. The combination of Collagen + MTA produced the most significant enhancement at both day 3 (Fig. 2a) and day 7 (Fig. 2b), indicating a synergistic interaction. Statistical analysis confirmed significant differences between treatment groups, with the combined treatment outperforming Collagen and MTA individually. By day 7, MTA maintained a higher response than Collagen, while the Collagen + MTA group continued to show the strongest osteogenic effect. Collagen’s impact was more pronounced at the early time point, supporting early matrix formation rather than sustained signaling. Overall, the combination of Collagen and MTA was the most effective in promoting DPSC mineralization at day 3 and day 7 (Fig. 2c), enhancing both early and sustained ALP activity.

**Table 2:** ALP activity and ARS of DPSCs at two-time intervals of exposure to ODM, Collagen, MTA, and Collagen + MTA

Variable	ODM	Collagen	MTA	Collagen + MTA	*p*-value
ALP (U/mL) at day 3	33.7 ± 1.27^b/c/d^	40.1 ± 2.0^a/c/d^	46.1 ± 2.1^b/d^	53.1 ± 1.9^a/b/c^	0.0001
ALP (U/mL) at day 3	38.0 ± 1.23^c/d^	42.8 ± 2.2^c/d^	51.8 ± 1.4^a/b/d^	60.7 ± 2.0^a/b/c^	0.0001
ARS (µM) at day 7	2.52 ± 0.30^c/d^	3.9 ± 0.65^c/d^	6.5 ± 1.2^a/d^	10.4 ± 2.1^a/b/c^	0.0003
ARS (µM) at day 14	5.34 ± 0.94^c/d^	8.7 ± 1.7^c/d^	16.0 ± 0.9^a/b/d^	22.6 ± 2.4^a/b/c^	0.0001

Mean ± SD values of ALP activity at day 3 and day 7 are presented for all experimental groups. One-way ANOVA showed significant differences across groups at both time points (*p* = 0.0001)

*Abbreviations*: *ODM* osteogenic differentiation medium; *MTA* mineral trioxide aggregate; *ALP* alkaline phosphatase, *DPSCs* Dental pulp-derived mesenchymal stem cells, *ARS* Alizarin Red S Concentration

Superscript letters denote statistically significant pairwise comparisons versus ODM (^a^), Collagen (^b^), MTA (^c^), and Collagen + MTA (^d^)

### Enhanced mineralization of DPSCs induced by MTA, collagen, and their synergistic combination (Table 2; Fig. 3)

**Fig. 3:**
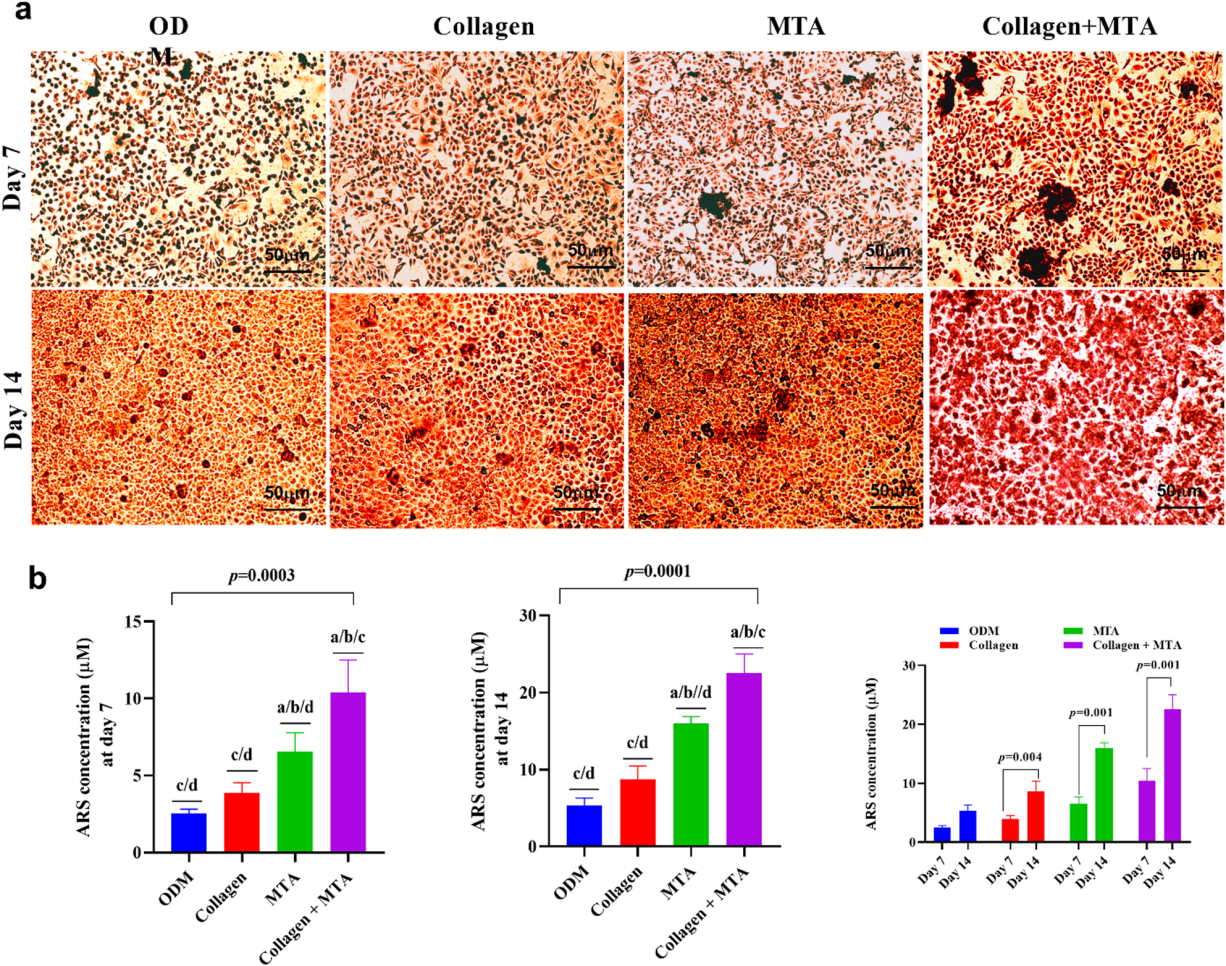
Alizarin Red S staining and ARS quantification of mineralized matrix in DPSCs cultured under different conditions for 7 and 14 days. (**a**) Representative images show calcium deposition in DPSCs cultured in osteogenic differentiation medium (ODM) alone (control), ODM supplemented with Collagen, MTA, or a combination of Collagen + MTA. The magnification is 20×, and the scale bar is 50 μm. Images were captured using the LABOMED Trinocular inverted phase contrast microscope model TCM400 and Atlas 16MP CMOS USB Camera with PixelPro 3.0 software (LABOMED, USA). **(b)** Bar graphs show ARS concentrations representing mineral deposition at days 7 and 14. The correlation between ARS concentration at day 7 and day 14 is also presented for each treatment. Statistical significance was determined by one-way ANOVA followed by Tukey’s post-hoc test. ^a^: statistical significance compared to cells cultured in DMEM (*p* > 0.05), ^b^: statistical significance compared to cells cultured in media supplemented with collagen (*p* > 0.05), ^c^: statistical significance compared to cells cultured media supplemented with MTA (*p* > 0.05), ^d^: statistical significance compared to cells cultured media supplemented with Collagen + MTA (*p* > 0.05)

The ARS quantification results demonstrated a clear, progressive enhancement in mineral deposition depending on the material used, with MTA showing significant calcium-rich matrix formation at day 7 compared to the osteogenic medium and Collagen. The combination of Collagen and MTA further amplified mineralization, indicating a synergistic effect that enhanced MTA’s osteoinductive properties. At day 14, the Collagen + MTA group continued to outperform all other groups, confirming its superior long-term mineralization capacity (Fig. 3a). Post-hoc analysis revealed that MTA alone was significantly more effective than Collagen, and the combination treatment was significantly higher than both individual materials. The sustained superiority of the Collagen + MTA group across both time points indicated enhanced maturation of mineralized nodules, with early mineral deposition predicting later mineral maturation (Fig. 3b). Overall, the ARS data show that MTA is more effective than Collagen alone, and the combination of Collagen and MTA provides the most potent and sustained mineralization stimulus, making it ideal for maximizing DPSC-mediated hard-tissue regeneration.

### Odontogenic gene expression profile of DPSCs induced by biomaterials (Table 3; Fig. 4)

**Fig. 4:**
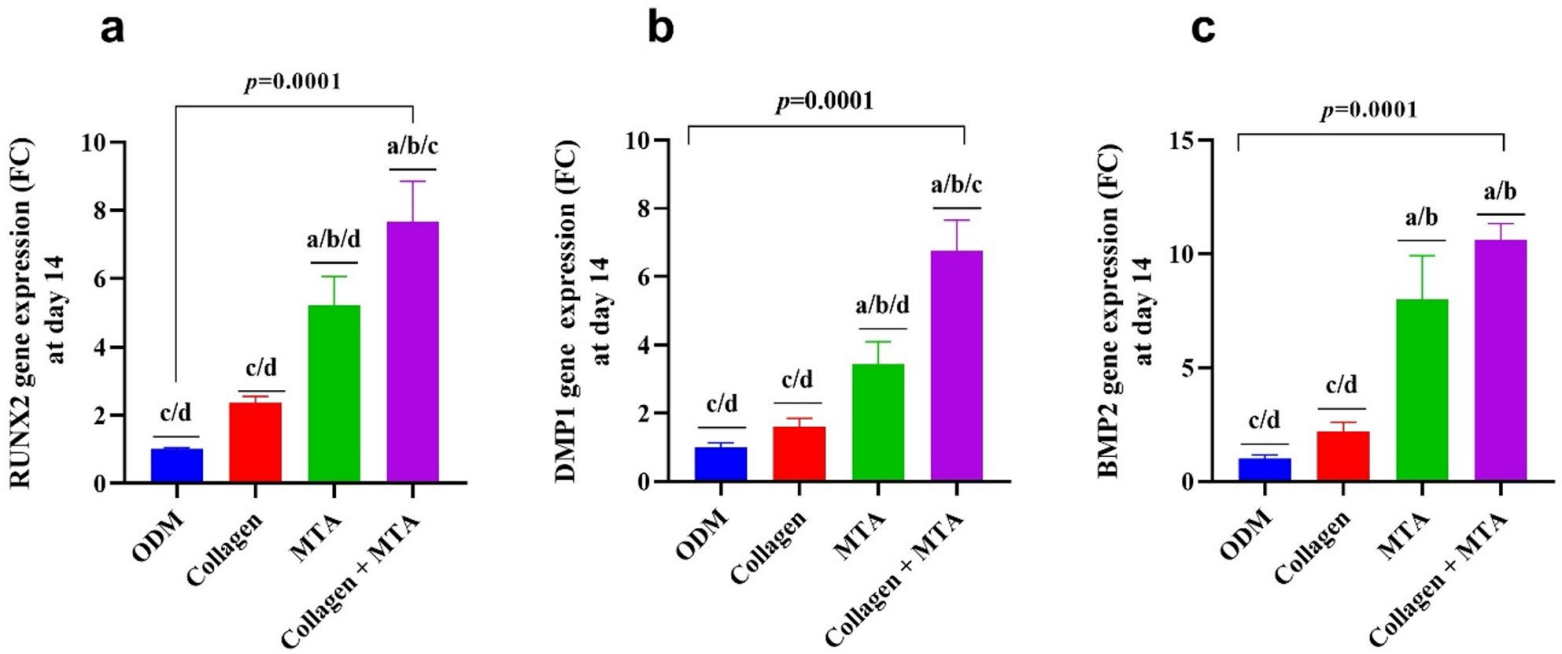
Odontogenic gene expression of DPSCs after 14 days of culture with ODM, Collagen, MTA, and Collagen + MTA. Bar graphs illustrate the fold-expression profiles of *RUNX2* (**a**), *DMP1* (**b**), and *BMP2***(c)**. The figure demonstrates a graded increase in odontogenic gene activation from ODM to Collagen to MTA, with Collagen + MTA producing the strongest overall transcriptional response. Statistical significance was determined using one-way ANOVA followed by Tukey’s post-hoc test. ^a^: statistical significance compared to cells cultured in DMEM (*p* > 0.05), ^b^: statistical significance compared to cells cultured in media supplemented with collagen (*p* > 0.05), ^c^: statistical significance compared to cells cultured media supplemented with MTA (*p* > 0.05), ^d^: statistical significance compared to cells cultured media supplemented with Collagen + MTA (*p* > 0.05)

After 14 days of exposure to biomaterials, the gene expression profile of DPSCs revealed significant material-dependent enhancement in odontogenic differentiation. Cells cultured with Collagen showed modest increases in odontogenic gene expression, indicating a supportive role. In contrast, MTA strongly upregulated the expression of key odontogenic genes, including *RUNX2***(**Fig. 4a), *DMP1***(**Fig. 4b), and *BMP2***(**Fig. 4c), highlighting its potent capacity to stimulate early transcription factors, dentin matrix proteins, and morphogenetic signals. The most striking results were observed in the Collagen + MTA group, which exhibited the highest expression levels across all genes, suggesting a synergistic effect where Collagen enhances the cellular response to MTA. Statistical analyses confirmed that the Collagen + MTA combination significantly outperformed all other groups, including MTA alone, in activating transcriptional regulators and matrix-forming genes. These findings demonstrate that Collagen supports transcriptional activity, MTA strongly induces odontogenic genes, and the Collagen + MTA combination produces the most robust and coordinated odontogenic gene expression, enhancing DPSC differentiation at the molecular level.

**Table 3:** Gene expression in DPSCs at day 14 of exposure to ODM, Collagen, MTA, and Collagen + MTA

	ODM	Collagen	MTA	Collagen + MTA	*p*-value
*RUNX2 (FC)*	1.0 ± 0.05^c/d^	2.4 ± 0.2^c/d^	5.2 ± 0.8^a/b/d^	7.7 ± 1.2^a/b/c^	0.0001
*DMP1* (FC)	1.01 ± 0.14^c/d^	1.6 ± 0.25^c/d^	3.5 ± 0.64^a/b/d^	6.8 ± 0.9^a/b/c^	0.0001
*BMP*2 (FC)	1.01 ± 0.16^c/d^	2.2 ± 0.4^c/d^	8.0 ± 1.9^a/b^	10.6 ± 0.7^a/b^	0.0001

Mean ± SD values of ALP activity at day 3 and day 7 are presented for all experimental groups. One-way ANOVA showed significant differences across groups at both time points (*p* = 0.0001)

*Abbreviations*: *ODM* osteogenic differentiation medium; *MTA *= mineral trioxide aggregate; *DPSCs* Dental pulp-derived mesenchymal stem cells, *RUNX2*: runt-related transcription factor 2, *DMP1*: dentin matrix acidic phosphoprotein 1, *BMP2*: bone morphogenetic protein 1

Superscript letters denote statistically significant pairwise comparisons versus ODM ^(a^), Collagen (^b^), MTA (^c^), and Collagen + MTA (^d^)

## Discussion

This study aimed to evaluate the effects of MTA and collagen, both separately and in combination, on the proliferative and odontogenic differentiation potential of hDPSCs. The results revealed significant differences in the proliferative response of DPSCs depending on the biomaterial used. While MTA alone caused a slight decrease in cell viability, collagen significantly increased cell viability to 116%, confirming its biocompatibility and pro-growth properties. Collagen’s ability to mimic the extracellular matrix (ECM) of dental pulp tissues promotes cell attachment [30], proliferation, and differentiation into odontoblast-like cells [31]. The collagen membrane, primarily composed of type I collagen, provides a favorable environment for stem cells, facilitates odontogenic differentiation, and stimulates angiogenesis, which is crucial for tissue regeneration [32].

The most striking result was observed with the combination of collagen and MTA, which exhibited the highest cell viability. This synergistic effect suggests that collagen may enhance MTA’s bioactivity, potentially by improving its delayed release profile and creating a more favorable environment for DPSC metabolic activity [33, 34]. This finding was supported by statistical analysis, which revealed significant differences between experimental groups, with Collagen + MTA outperforming all other treatments.

Regarding odontogenic differentiation, collagen alone caused a modest increase in alkaline phosphatase (ALP) activity, while MTA induced a stronger stimulatory effect, consistent with its well-known osteoinductive capacity [35]. The combination of collagen and MTA resulted in the most significant enhancement of ALP activity at both day 3 and day 7, suggesting a synergistic interaction between the two materials. This synergy was also reflected in the mineralization assay, where MTA alone induced significant calcium-rich matrix production by day 7, but the combination of collagen and MTA produced the most substantial mineralization. This suggests that collagen enhances MTA’s biological activity [36], rather than simply contributing a minor effect.

At the later time point (day 14), the combination of collagen membrane and MTA conditioned continued to outperform all other treatments, indicating enhanced long-term mineralization potential. Given that hDPSCs were exposed to conditioned media rather than cultured directly on the materials, the observed effects are most likely mediated by soluble bioactive factors released from the collagen membrane and MTA. In this context, collagen may contribute indirectly by modulating the biological microenvironment, potentially influencing the availability, stability, or release kinetics of mineralization-related ions and signaling molecules derived from MTA [37]. Additionally, collagen -derived components present in the conditioned medium may facilitates the release or preservation of growth factors and extracellular matrix-associated proteins, that support odontogenic differentiation and mineralization processes in DPSCs [38]. Collectively, these findings suggest that the enhanced mineralization observed in the combined treatment group is driven by biochemical interactions between collagen- and MTA-derived factors, rather than by direct scaffold-mediated cell interactions [37].

Collagen membrane–conditioned medium alone induced a modest upregulation of odontogenic-associated genes (RUNX2, DMP1, and BMP2), whereas MTA-conditioned medium produced a more pronounced increase in their expression. The highest levels of gene upregulation were observed in the combined collagen membrane + MTA–conditioned medium group, indicating a synergistic effect on odontogenic differentiation. Given that cells were exposed to conditioned media rather than direct material contact, this synergy is more likely attributable to interactions between soluble bioactive factors released from collagen membrane and MTA, rather than to a physical ECM scaffold effect. Collagen-derived components present in the conditioned medium may modulate the cellular microenvironment and enhance cellular responsiveness to MTA-derived signaling molecules, thereby promoting the activation of key transcriptional regulators involved in odontogenic differentiation, including *RUNX2*, *DMP1*, and *BMP2*. These findings suggest that the complementary effects of collagen membrane and MTA on gene expression are mediated through biochemical signaling pathways rather than direct scaffold-mediated mechanisms.

MTA’s ability to upregulate key odontogenic genes, including RUNX2 and BMP2, is consistent with previous studies demonstrating its osteoinductive properties [39]. MTA has been shown to stimulate the expression of transcription factors involved in dentinogenesis [40], such as RUNX2, and to promote the synthesis of BMP2, a protein essential for odontogenic differentiation and mineralization [41, 42].

Despite its well-documented bioactivity and odontogenic potential, mineral trioxide aggregate (MTA) presents several practical and biological limitations, including difficult handling properties, prolonged setting time, tooth discoloration, and concerns related to its high alkalinity, which may affect cellular responses [43]. In addition, the presence of trace components such as bismuth oxide and aluminum-containing phases derived from Portland cement has raised questions regarding long-term biocompatibility. These limitations underscore the rationale for exploring combination strategies, such as incorporating collagen-based materials, to modulate MTA-associated effects and potentially improve its biological performance. Within this context, the present study investigates whether combining collagen membrane with MTA—through soluble bioactive factors can enhance odontogenic outcomes while addressing some of the inherent drawbacks of MTA when used alone [44].

This in vitro study has some limitations, including potential variations in cell growth conditions that could affect the outcomes. Although variability in cell growth conditions is an inherent limitation of in vitro studies, this was minimized by using standardized culture protocols, defined passage numbers, and biological replicates across all experimental groups. Future studies may further address this limitation by employing donor-matched cell populations, controlled material release systems, and direct cell–material interaction models to enhance reproducibility and translational relevance. Additionally, the study may not fully replicate the complexity of in vivo environments, as it lacks direct interaction with biological tissues. While alternative methodologies are still being validated, they may delay their implementation in medical device testing. Despite these limitations, in vitro testing offers significant advantages, such as providing a controlled environment to study biological responses to biomaterials, reducing the need for animal testing, and accelerating the development process. Furthermore, in vitro approaches follow internationally accepted guidelines, ensuring consistency and reliability in the results.

In conclusion, collagen-conditioned medium alone exhibited limited inductive effects on odontogenic differentiation, whereas MTA-conditioned medium significantly enhanced odontogenic gene expression and differentiation of hDPSCs. The combined collagen membrane + MTA–conditioned medium produced the most pronounced and consistent biological responses, indicating a synergistic effect mediated by soluble bioactive factors rather than direct material–cell interactions. These findings support the potential of combining collagen-based materials with MTA to modulate odontogenic differentiation in vitro. Further studies are warranted to elucidate the underlying molecular mechanisms and to evaluate optimized material delivery strategies and direct interaction models for future dental pulp regenerative applications.

## Data Availability

The data supporting this study’s findings are available from the corresponding author upon reasonable request.

## Abbreviations

hDPSCs: Human Dental Pulp Stem Cells
MTA: Mineral Trioxide Aggregate
ALP: Alkaline Phosphatase
ARS: Alizarin Red S
MTA: Mineral Trioxide Aggregate
ECM: Extracellular Matrix
ODM: Osteogenic Differentiation Medium
PBS: Phosphate-Buffered Saline
FBS: Fetal Bovine Serum
PSA: Penicillin-Streptomycin-Amphotericin B
ODM: Osteogenic Differentiation MediumNot applicable
